# Structural abnormalities of the elbow joint in high school baseball players exposed to the COVID-19 pandemic during their elementary and junior high school years: a sonographic analysis of 354 players at high school entry

**DOI:** 10.1016/j.jseint.2025.101605

**Published:** 2025-12-24

**Authors:** Ryosuke Nishi, Takahisa Ogawa, Yuki Obokata, Atushi Kubota, Masashi Aoyagi, Kozo Furushima, Kunio Kamatani

**Affiliations:** a Rehabilitation Center, East Maebashi Orthopedic Hospital, Maebashi, Gunma, Japan; b Graduate School of Health and Sports Science, Juntendo University, Chiba, Japan; c Macquarie University Australian Institute of Health Innovation (AIHI), Sydney, Australia; d Japan Institute of Sports Sciences, Tokyo, Japan; e Department of Orthopedic Surgery, Keiyu Orthopedic Hospital, Tatebayashi, Gunma, Japan; f Department of Orthopedic Surgery, East Maebashi Orthopedic Hospital, Maebashi, Gunma, Japan

**Keywords:** COVID-19, Elbow, Medial epicondyle, Ulnar collateral ligament, Baseball, Throwing load management

## Abstract

**Background and Hypothesis:**

Previous studies have shown that increased pitching load during growth is associated with medial epicondyle hypertrophy and ulnar collateral ligament thickening. The COVID-19 pandemic caused prolonged restrictions on sports activities among youth, especially during their elementary and junior high school years. We hypothesized that pandemic-related reductions in pitching opportunities would result in a lower prevalence of these structural adaptations in high school baseball players.

**Methods:**

Of the 354 high school baseball players enrolled between 2015 and 2025, 119 were pitchers and 235 were fielders. All participants had undergone ultrasound screening at the time of high school enrollment. We categorized them into a Pandemic group (entered high school between 2021 and 2025) and a pre-pandemic group (entered high school in 2020 or earlier). Medial elbow structures were assessed for 5 abnormalities: hypertrophy, irregularity, bone fragment, hypoechoic findings, and thickening. Group differences were analyzed using chi-square tests and odds ratios. Stepwise multivariable logistic regression was conducted to identify independent factors associated with medial epicondyle hypertrophy.

**Results:**

The Pandemic group demonstrated a significantly higher prevalence of medial epicondyle hypertrophy compared with the pre-pandemic group (41.1% vs. 28.3%, *P* = .01), as well as a significantly lower prevalence of ulnar collateral ligament thickening (51.5% vs. 67.5%, *P* = .01) and hypoechoic findings (23.9% vs. 34.5%, *P* = .01). Stepwise logistic regression identified membership of the Pandemic group as the sole independent predictor of medial epicondyle hypertrophy (OR = 1.78, 95% CI: 1.14–2.78, *P* = .01).

**Discussion and Conclusion:**

High school baseball players who experienced the COVID-19 pandemic during crucial growth periods exhibited a paradoxical increase in medial epicondyle hypertrophy along with a decrease in ligamentous abnormalities. Supplemental questionnaire data from a subset of participants suggested that abrupt increases in pitching volume after restrictions, rather than total cumulative load, may have contributed to hypertrophy. These findings indicate that sudden changes in throwing load and inadequate physiological adaptation during pandemic-related restrictions may have disrupted normal remodeling processes in the developing elbow. To prevent elbow abnormalities in growing baseball players, comprehensive throwing load management—including frequency and intensity, not just pitch count—is essential, with particular attention to gradual return-to-throwing programs after prolonged inactivity.

Upper extremity injuries associated with overhead throwing motions, particularly those involving the shoulder and elbow, are commonly observed in baseball and pose a significant risk to the athletic careers of players. Throwing-related elbow injuries are a particular concern for young baseball players, not only among pitchers but also among fielders. Lyman et al^13^ and Robb et al^23^ reported that the number of pitches thrown, physical factors such as joint range of motion and muscle strength, and improper throwing mechanics all contribute to the development of pitching-related injuries.

The pitching motion is typically divided into 6 phases: wind-up, early cocking, late cocking, acceleration, deceleration, and follow-through. Maximum external rotation, which represents the transitional phase between late cocking and acceleration, has been associated with an increased risk of elbow injury. During maximum external rotation, a valgus torque of approximately 64 N m is generated at the elbow joint.^7^ Repeated exposure to this extreme valgus torque at the ulnar collateral ligament (UCL) is considered a major factor in the development of elbow injuries.^15^ Indeed, Anz et al^2^ identified valgus torque at the elbow joint as the sole mechanical cause of such injuries, as it can damage the ligament, muscle, and joint structures that constitute the medial stabilizing mechanism of the elbow. These injuries result from the cumulative mechanical stress associated with various types of throwing, including pitching and fielding activities.

In Japan, elbow injuries are most commonly observed among school-aged children, with surgical intervention often required during adolescence. These elbow injuries have been found to result in structural abnormalities of the medial epicondyle of the humerus.^29^ The medial epicondyle is a bony prominence located on the inner aspect of the elbow, serving as the attachment site for the forearm flexor and pronator muscles. Repeated throwing motions during school-age years may exert traction forces on the medial aspect of the elbow, potentially causing medial epicondyle hypertrophy, bone fragments, and irregularity.^9^ This repetitive stress has also been found to cause thickening of the UCL.^30^ Previous studies have suggested that increased pitching volume is associated with a higher prevalence of both medial epicondyle hypertrophy and UCL thickening.^14^^,^^17^^,^^26^ Furthermore, we reported in earlier research that hypertrophy and irregularities of the medial epicondyle are risk factors for the subsequent development of throwing-related elbow injuries.^18^

From 2020 onward, the COVID-19 pandemic severely limited opportunities for elementary and junior high school students in Japan to engage in progressive throwing activities, including both pitching and fielding.^12^ Previous studies have reported that injury rates significantly decreased during COVID-19 lockdowns but increased markedly following the pandemic, probably due to a sudden return to high-intensity activities without sufficient conditioning and preparation.^31^ However, despite fluctuations in injury rates, no significant changes were observed in the anatomical or demographic distribution of injuries. This suggests that although the types of injuries remained consistent, their frequency increased following the resumption of athletic activity.^19^ To date, no studies have definitively examined the mechanisms by which the effects of the COVID-19 pandemic affected the structural adaptation of the elbow joint and the risk of developing disability among baseball players, especially those who were still growing. Thus, it remains unclear how the restrictions imposed by the pandemic on pitching-related stimuli affected the musculoskeletal development of these players. It is possible that athletes returned to competitive throwing with abrupt increases in intensity, frequency, and throwing volume following this prolonged period of limited mechanical stimulation. Based on previous findings, we hypothesized that pandemic-related reductions in pitching volume during the growth period would result in a lower prevalence of medial epicondyle hypertrophy and UCL thickening compared with players from the pre-pandemic period.

To investigate the association between the effects of the COVID-19 pandemic and structural abnormalities of the elbow joint, we examined the prevalence of medial epicondyle and UCL abnormalities among high school baseball players who experienced the pandemic during their growth period and compared the results with those of players from the pre-pandemic period.

## Materials and methods

We conducted a cross-sectional study to investigate the prevalence of medial epicondyle and UCL structural abnormalities in high school baseball players affected by the COVID-19 pandemic during their growth years. The study was approved by the institutional review board (approval number: 2024-1), and written informed consent was obtained from all participants and their parents or legal guardians.

### Participants

A survey was administered to newly enrolled members of a nationally competitive high school baseball team between 2015 and 2025. Based on the results, players who met the eligibility criteria were selected for inclusion in the study. The inclusion criteria were: (1) giving informed consent, (2) having played baseball during both elementary and junior high school, and (3) being able to undergo ultrasound evaluation of the elbow joint immediately after entering high school. The exclusion criteria were those who did not give consent, those who had never played baseball in elementary and junior high school, those who did not participate in practice on the day of the examination, those who were unable to undergo an ultrasound examination, and those who were prohibited from sports activities by a doctor due to surgery.

Participants completed a questionnaire on height, weight, years of baseball experience, playing position, age, throwing arm, current medical conditions, past medical history, surgical history, and any physician restrictions on sports.

### Structural abnormality of the elbow joint

To evaluate structural abnormalities of the elbow joint, an ultrasound system was used to assess the UCL and the medial epicondyle. This was the same ultrasound protocol used for research on the medial epicondyle and UCL reported previously by our group.^18^ Each participant was assessed in a sitting position with the forearm in the mid-rotational position and the elbow flexed approximately 90°, while the shoulder was in mild external rotation (with the elbow placed on a table). Using an ultrasound system (SONIMAGE, Konica Minolta, Tokyo), a physical therapist (R.N.) placed the linear probe on the medial side of the elbow joint using the medial humeral condyle as a landmark and collected static sonographic images of the UCL. The focus point was set at the UCL, the frequency was 11 MHz, and the contrast was adjusted to clearly display the UCL. A board-certified elbow specialist (K.F.) with 27 years of clinical experience interpreted all images and, based on these, diagnosed thickening and damage of the UCL (hypoechoic findings) as well as abnormal bone morphology of the medial epicondyle of the humerus (irregular images, bone fragments, hypertrophy).^3^^,^^16^^,^^24^^,^^29^^,^^30^

**Figure 1:**
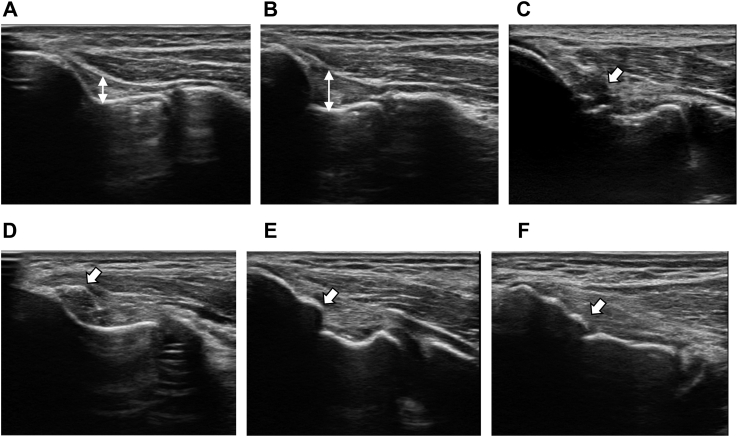
Structural abnormalities of the elbow joint. (**A**) Nondominant side; (**B**) thickening; (**C**) lesion (hypoechoic finding); (**D**) bone fragments; (**E**) hypertrophy; (**F**) irregularity.

The morphological status of the medial epicondyle and UCL were evaluated using longitudinal images, and positive findings were obtained only on the throwing side. Thickening of the UCL was evaluated through comparison with the nondominant side and defined as a visually obvious difference referred to as the “large area” (see Fig. 1). The physical therapist who performed the ultrasound system had more than 10 years of experience in using ultrasound to examine the elbow joints of baseball players.

Ultrasound images of the medial aspect of the elbow were then obtained. Morphological changes in the UCL and the distal aspect of the medial epicondyle are displayed in Fig. 1. Arrows indicate structural abnormalities. A two-headed arrow illustrates the side-to-side difference in UCL thickness between the dominant and nondominant arms, thereby identifying thickening.

### Statistical analysis

Participants were stratified based on their year of high school entry. New recruits who entered high school between 2021 and 2025 were defined as the “Pandemic group,” as it was assumed that they had been affected by the COVID-19 pandemic during their elementary and junior high school years. Players who entered high school between 2015 and 2020 were defined as the “pre-pandemic group.”

To examine whether the COVID-19 pandemic was associated with abnormalities of the medial epicondyle and the UCL, differences in categorical variables between the 2 groups were assessed using the chi-square test or Fisher exact test. We also calculated odds ratios (ORs) and 95% confidence intervals (CIs) to identify associations between the groups. For continuous variables, independent *t*-tests were performed. In addition, a stepwise multivariable logistic regression analysis based on the Akaike Information Criterion^1^ was performed to identify factors associated with medial epicondyle hypertrophy. The dependent variable was the presence or absence of medial epicondyle hypertrophy in the elbow joint. The independent variables were height, weight, years of baseball experience, COVID-19 pandemic exposure, history of elbow injuries during elementary and junior high school years, and playing position. All statistical analyses were performed using R version 4.0.2 and the significance level was set at *P* < .05.

### Supplementary questionnaire survey

In addition, in the Pandemic group, we conducted a supplemental questionnaire survey after the initial data collection to assess changes in training and pitching load following the COVID-19 pandemic. This survey targeted a subset of participants with and without medial epicondyle abnormalities detected on ultrasound. The questionnaire inquired about changes in (1) total practice volume, (2) number of pitches, (3) pitching frequency, (4) interval between pitching sessions, and (5) pitching intensity after the resumption of activities. For each item, responses were recorded on a 5-point scale: “increased,” “slightly increased,” “no change,” “slightly decreased,” or “decreased.” Participation in this supplementary survey was voluntary.

## Results

**Figure 2:**
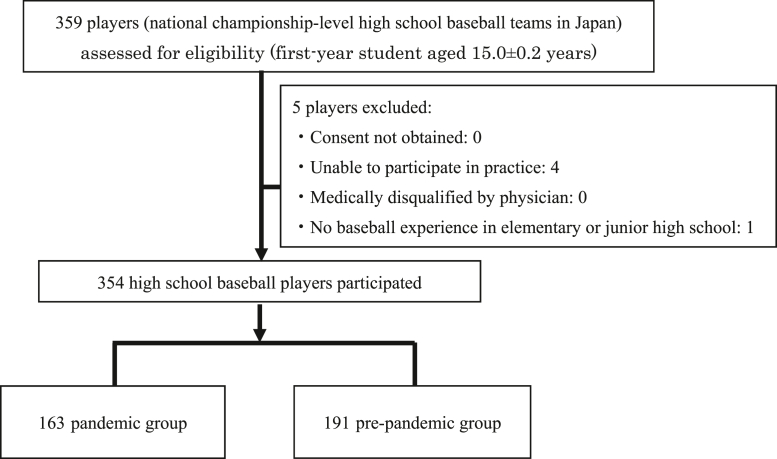
Flow diagram of participant enrollment, exclusions, and group allocation. Pre-pandemic group entered high school between 2015-2020. Pandemic group entered high school between 2021-2025.

The Pandemic group included 163 students who enrolled between 2021 and 2025, while the pre-pandemic group consisted of 191 students who enrolled between 2015 and 2020 (Fig. 2). A detailed breakdown of all participants is presented in Table I.

**Table I:** Descriptive characteristics and ultrasound findings for all participants, pitchers, and fielders.∗

Variables	All participants (N = 354)	Pitchers (N = 119)	Fielders (N = 235)
Height, cm	173.0 ± 5.4	174.4 ± 5.6	171.0 ± 5.6
Weight, kg	69.6 ± 8.2	71.4 ± 7.5	68.7 ± 8.4
Years of baseball experience, yr	7.2 ± 1.5	7.2 ± 1.7	7.2 ± 1.4
UCL			
Thickening	213 (60.1%)	75 (63.0%)	138 (58.7%)
Hypoechoic finding	105 (29.6%)	35 (29.4%)	70 (29.7%)
Medial epicondyle			
Irregularity	127 (35.8%)	45 (37.8%)	82 (34.8%)
Bone fragments	68 (19.2%)	29 (24.3%)	39 (16.6%)
Hypertrophy	121 (34.1%)	42 (35.2%)	79 (33.6%)
Past medical history			
Elbow	171 (48.3%)	57 (47.8%)	114 (48.5%)
Shoulder	148 (41.8%)	46 (38.6%)	102 (43.4%)

*UCL*, ulnar collateral ligament; *SD*, standard deviation.

∗ Categorical variables are expressed as n (%), and continuous variables are shown as mean ± SD. Values indicate the number (%) of participants with ultrasound-identified abnormalities in the UCL, and medial epicondyle. All data reflect dominant-side findings only.

Overall, the Pandemic group had significantly higher body weight and a fewer years of baseball experience compared with the pre-pandemic group. No significant difference in height was observed. Medial epicondyle hypertrophy was significantly more prevalent in the Pandemic group (OR = 1.77, 95% CI: 1.15-2.73, *P* = .01), whereas ligament thickening (OR = 0.60, 95% CI: 0.41-0.88, *P* = .01) and hypoechoic findings (OR = 0.51, 95% CI: 0.31-0.83, *P* = .01) were more frequently observed in the pre-pandemic group. No significant differences were observed in the prevalence of bone fragments, irregular findings, or medical history between the groups (Table II).

**Table II:** Group comparison of structural abnormalities (pandemic group vs. pre-pandemic group).∗

Variables	Pandemic group (N = 163)	Pre-pandemic group (N = 191)	*P* value	Odds ratio	95% CI
Height, cm	172.8 ± 5.9	172.2 ± 5.0	.19		
Weight, kg	71.9 ± 8.5	67.6 ± 7.4	**.01**		
Years of baseball experience, yr	6.9 ± 1.6	7.3 ± 1.4	**.01**		
UCL					
Thickening	84 (51.5%)	129 (67.5%)	**.01**	0.60	0.37-0.95
Hypoechoic finding	39 (23.9%)	66 (34.5%)	**.01**	0.51	0.33-0.79
Medial epicondyle					
Irregularity	59 (36.1%)	68 (35.6%)	.90	1.03	0.66-1.59
Bone fragments	33 (20.2%)	35 (18.3%)	.64	1.13	0.67-1.92
Hypertrophy	67 (41.1%)	54 (28.2%)	**.01**	1.77	1.44-2.76
Elbow history					
Elementary school	73 (44.7%)	98 (51.3%)	.22	0.77	0.51-1.17
Junior high school	68 (41.7%)	80 (41.8%)	.97	1.1	0.71-1.83

*UCL*, ulnar collateral ligament; *CI*, confidence interval; *SD*, standard deviation.

Bold values indicate statistically significant differences (*P* < .05).

∗ Categorical variables are expressed as n (%), and continuous variables are shown as mean ± SD. Values indicate the number (%) of participants with ultrasound-identified abnormalities in the UCL, and medial epicondyle. Statistical significance was determined using an unpaired *t*-test or chi-square test. All data reflect dominant-side findings only.

In the pitcher-only analysis, the Pandemic group (n = 58) had significantly higher body weight and fewer years of baseball experience compared with the pre-pandemic group (n = 61), which is consistent with the findings from the overall cohort. UCL thickening (OR = 0.24, 95% CI: 0.09-0.61, *P* = .01) and hypoechoic findings (OR = 0.43, 95% CI: 0.19-0.95, *P* = .04) were significantly more prevalent in the pre-pandemic group. No significant differences in medial epicondyle abnormalities or history of elbow injury were observed (Table III).

**Table III:** Comparison of ultrasound findings for pitchers: pandemic vs. pre-pandemic group.∗

Variables	Pandemic group (N = 58)	pre-pandemic group (N = 61)	*P* value	Odds ratio	95% CI
Height, cm	174.8 ± 6.1	173.8 ± 5.1	.46		
Weight, kg	73.0 ± 7.8	69.8 ± 6.9	**.04**		
Years of baseball experience, yr	6.7 ± 1.8	7.6 ± 1.5	**.01**		
UCL					
Thickening	27 (46.6%)	48 (78.7%)	**.01**	0.24	0.11-0.53
Hypoechoic finding	12 (20.7%)	23 (37.7%)	**.04**	0.43	0.19-0.98
Medial epicondyle					
Irregularity	22 (37.9%)	23 (37.7%)	1.00	1.01	0.48-2.12
Bone fragments	12 (20.7%)	17 (27.8%)	.39	0.68	0.29-1.57
Hypertrophy	20 (34.5%)	22 (36.1%)	1.00	0.93	0.44-1.98
Elbow history					
Elementary school	25 (43.1%)	32 (52.5%)	.36	0.69	0.33-1.41
Junior high school	23 (39.7%)	23 (37.7%)	.85	1.09	0.52-2.27

*UCL*, ulnar collateral ligament; *CI*, confidence interval; *SD*, standard deviation.

Bold values indicate statistically significant differences (*P* < .05).

∗ Categorical variables are expressed as n (%) and continuous variables are shown as mean ± SD. Values indicate the number (%) of pitchers with ultrasound-identified abnormalities in the UCL, and medial epicondyle. Statistical significance was determined using an unpaired *t*-test or chi-square test. All data reflect dominant-side findings only.

Similarly, in the fielders-only analysis, the Pandemic group (n = 105) had significantly higher body weight and fewer years of baseball experience compared with the pre-pandemic group (n = 130). Among medial epicondyle abnormalities, hypertrophy was significantly more prevalent in the Pandemic group (OR = 2.48, 95% CI: 1.23-4.99, *P* = .01). No significant differences were observed in bone fragments, irregularities, UCL abnormalities, or history of elbow injury (Table IV).

**Table IV:** Comparison of ultrasound findings for fielders: pandemic vs. pre-pandemic group.∗

Variables	Pandemic group (N = 105)	Pre-pandemic group (N = 130)	*P* value	Odds ratio	95% CI
Height, cm	171.6 ± 5.5	171.5 ± 4.8	.78		
Weight, kg	71.4 ± 8.9	66.5 ± 7.4	**.01**		
Years of baseball experience, yr	7.0 ± 1.5	7.4 ± 1.4	**.04**		
UCL					
Thickening	57 (54.3%)	81 (62.3%)	.23	0.72	0.43-1.21
Hypoechoic finding	27 (25.7%)	43 (33.1%)	.25	0.70	0.40-1.24
Medial epicondyle					
Irregularity	37 (35.2%)	45 (34.6%)	1.00	1.03	0.60-1.76
Bone fragments	21 (20.0%)	18 (13.8%)	.20	1.56	0.78-3.10
Hypertrophy	47 (44.8%)	32 (24.6%)	**.01**	2.48	1.43-4.32
Elbow history					
Elementary school	48 (45.7%)	66 (50.8%)	.51	0.82	0.49-1.37
Junior high school	45 (42.9%)	57 (43.8%)	.89	0.96	0.57-1.61

*UCL*, ulnar collateral ligament; *CI*, confidence interval; *SD*, standard deviation.

Bold values indicate statistically significant differences (*P* < .05).

∗ Categorical variables are expressed as n (%) and continuous variables are shown as mean ± SD. Values indicate the number (%) of fielders with ultrasound-identified abnormalities in the UCL, and medial epicondyle. Statistical significance was determined using an unpaired *t*-test or chi-square test. All data reflect dominant-side findings only.

A stepwise multivariable logistic regression analysis based on the Akaike Information Criterion was performed to identify factors associated with medial epicondyle hypertrophy. The dependent variable was the presence or absence of medial epicondyle hypertrophy. The independent variables were height, weight, years of baseball experience, playing position, history of elbow injuries during elementary and junior high school, and experience of the COVID-19 pandemic. Spending one's elementary and junior high school years during the COVID-19 pandemic was identified as the only significant factor associated with medial epicondyle hypertrophy (OR = 1.78, 95% CI: 1.14-2.78, *P* = .01). History of elbow injuries during junior high school was included in the model but was not significant (*P* = .08). Position, height, weight, years of baseball experience, and history of elbow injuries during elementary school were not statistically significant predictors (*P* = .54, .48, .51, .75, and .38, respectively) (Table V).

**Table V:** Multivariable logistic regression for predictors of medial epicondyle hypertrophy.∗

Variables	Estimate	*P* value	Standard error	Odds ratio	95% CI	VIF
Pandemic group	0.57	.01	0.19	1.78	1.14-2.78	1.00
Junior high school elbow history	0.38	.08	0.22	1.47	0.94-2.31	1.00
(Intercept)	−1.10	.01	0.19			

*CI*, confidence interval; *VIF*, variance inflation factor.

Model X^2^ test: *P* < .05.

Hosmer–Lemeshow test: *P* = .99.

Probability of the success rate: 65.8%.

Pandemic group = 1, pre-pandemic group = 0.

Position: pitcher = 1, fielders = 0.

Elementary school elbow history: with injury = 1, without injury = 0.

Junior high school elbow history: with injury = 1, without injury = 0.

∗ Multivariable model adjusted for height, weight, years of experience, position, and elbow injury history.

In the supplementary questionnaire administered to a subset of players in the Pandemic group, all players (100%) who reported a decrease in practice volume during the COVID-19 restrictions (“slightly decreased” or “decreased”) exhibited medial epicondyle hypertrophy, whereas none of the players (0%) who reported a decrease were without hypertrophy. Moreover, when examining changes in pitching parameters after the restrictions were lifted, hypertrophy was observed in 100% of players who reported increases in pitching intensity or shorter intervals between pitching sessions, while those who reported increases in pitching frequency or total pitch count alone showed a hypertrophy rate of 0%.

## Discussion

In this cross-sectional study, we investigated the prevalence of medial epicondyle bony morphology and UCL abnormalities among high school baseball players who attended elementary and junior high school during the COVID-19 pandemic. We also examined the factors associated with these structural abnormalities. Contrary to our initial hypothesis—that reduced pitching during the pandemic would lower the prevalence of structural changes—we found no such decrease, further contradicting our expectations.

Hypertrophy of the medial epicondyle and thickening of the UCL are considered to be adaptive responses, reflecting repetitive stress and the remodeling process of the medial elbow.^5^^,^^14^ Before the COVID-19 pandemic, the prevalence of bony morphological abnormalities in Japanese school-aged baseball players was reported to be 37.5%.^11^ In the present study, the prevalence of hypertrophy (34.1%), bone fragments (19.2%), and irregularities (35.8%) did not substantially differ from previous findings.

We identified a significantly higher prevalence of medial epicondyle hypertrophy in high school baseball players who experienced the COVID-19 pandemic during their elementary and junior high school years (41.1% vs. 28.3%, *P* = .01). Furthermore, logistic regression analysis identified experiencing the COVID-19 pandemic during the growth period as the only independent risk factor for hypertrophy (*P* = .01, OR = 1.78, 95% CI: 1.14-2.78). Among pitchers, no significant difference was observed between the 2 groups, whereas fielders demonstrated a marked increase in hypertrophy during the pandemic period. This suggests that medial epicondyle hypertrophy was not merely the result of physiological growth but may have been influenced by environmental factors—specifically, the restriction of sports activities during the pandemic and the subsequent abrupt resumption of physical activity, which may have influenced normal bone remodeling processes. From a clinical perspective, these findings underscore that gradual load management during the return-to-play phase is essential not only to prevent acute injuries but also to mitigate maladaptive bone remodeling, even in athletes with lower baseline throwing demands.

An unexpected finding of this study was that medial epicondyle hypertrophy was more frequently observed in fielders than in pitchers within the Pandemic group. Pitchers are generally considered to experience higher and more repetitive throwing loads; however, during the COVID-19–related activity restrictions, their training routines may have remained relatively structured compared with those of fielders. In contrast, many fielders likely had a substantial reduction in throwing exposure followed by a sudden increase in workload when regular activities resumed. Such fluctuations in throwing volume and frequency may have placed additional mechanical demands on the immature elbow, contributing to the observed structural adaptations. Although these interpretations remain speculative, the present findings suggest that workload management after periods of prolonged inactivity may be important not only for pitchers but also for fielders.

During the growth period, bone tissue is known to undergo structural remodeling in response to mechanical stimuli in accordance with Wolff's law.^8^ However, activity restrictions may have disrupted this stimulation, and the abrupt reloading upon the resumption of pitching may be related to an exaggerated remodeling response, leading to medial epicondyle hypertrophy in a greater number of players. Indeed, previous studies have reported a marked increase in the incidence of sports-related injuries following the COVID-19 pandemic.^31^ In addition, we previously demonstrated that hypertrophy of the medial epicondyle is a significant risk factor for subsequent throwing-related elbow injuries.^18^ Although the causal relationship remains uncertain, our supplementary questionnaire supports the possibility that the abrupt resumption of high-intensity throwing following prolonged inactivity contributed to the observed hypertrophy. Collectively,^18^^,^^31^ these findings suggest that throwing load management during the growth period should not rely solely on pitch count but must consider the frequency, intensity, and throwing volume of all throwing activities, including both pitching and fielding, particularly in the context of a progressive reintroduction of load. Such a comprehensive approach is critical for promoting appropriate musculoskeletal adaptation and minimizing the future risk of injuries in developing athletes.

The supplementary questionnaire was completed by 14 athletes (7 pitchers and 7 fielders), selected to include a balanced distribution of medial epicondyle hypertrophy and UCL thickening. Although limited in size, this subgroup provided contextual information regarding changes in throwing volume, frequency, and intensity after prolonged inactivity. Although the supplementary questionnaire was limited to a subset of the Pandemic group, the responses provide contextual evidence supporting the mechanism of abrupt load escalation after prolonged inactivity. Even though comparable data were not available for the pre-pandemic group, previous literature consistently shows that abrupt spikes in workload are a major risk factor for injury across youth sports. Thus, these findings align with established physiological principles and strengthen the plausibility of our interpretation.

In recent years, attention has increasingly focused on the Acute Chronic Workload Ratio as a framework for understanding injury risk in youth athletes.^22^^,^^25^^,^^28^ This metric captures fluctuations in workload by comparing short-term load (eg, 1 week) with medium-term load (eg, the 4-week average). Previous studies have suggested that increases or decreases in Acute Chronic Workload Ratio of approximately 33% may elevate the risk of injury.^25^ In baseball players, throwing exposure, including the number of pitches and innings pitched, has been associated with pitching-related injuries.^6^^,^^20^^,^^24^ However, pitch counts alone may not fully reflect the mechanical load placed on the upper extremity. In the present study, pitchers did not demonstrate a higher prevalence of medial epicondyle hypertrophy in the Pandemic group compared with the pre-pandemic group. One possible explanation is that structural stress may be determined not only by cumulative pitch volume but also by throwing frequency, recovery intervals, and the intensity of individual throws. Although workload metrics were not directly recorded, the findings imply that elbow adaptation may be shaped by multiple dimensions of load beyond pitch counts alone. Importantly, fluctuations in workload are not limited to pandemic-related activity restrictions; similar conditions arise after injury-related layoff or seasonal breaks. A more comprehensive approach to workload monitoring, integrating both volume and intensity, may therefore be essential when young athletes return to throwing after prolonged reductions in activity.

Among pitchers, thickening and hypoechoic changes were significantly more prevalent in the pre-pandemic group than in the Pandemic group (78.7% vs. 46.6% and 37.7% vs. 20.7%, respectively). Ciccotti et al^5^ reported that such ultrasonographic changes, along with ligamentous laxity, are common in the UCL of the throwing arm, reflecting adaptations to long-term repetitive stress. Similarly, Sutterer et al^27^ noted that UCL laxity represents remodeling in response to chronic loading. These findings suggest that in the pre-pandemic group, continuous pitching loads promoted structural remodeling of the ligament, which may include adaptive changes such as fibrosis, degeneration, and reduced vascularity.

Taken together, the results of this study suggest that bones and ligaments may exhibit distinct mechanisms of adaptation. Specifically, it is assumed that bone exhibits an exaggerated remodeling response triggered by abrupt reloading following a period of mechanical unloading, whereas ligaments are thought to undergo degeneration as a consequence of prolonged, chronic mechanical stress. Previous studies have reported that mechanical stress induced by exercise promotes bone remodeling in children.^4^ In this study, logistic regression analysis revealed that factors previously reported to be significant, such as player position,^6^ years of playing experience,^10^ and a history of elbow injuries,^21^ were not significantly associated with medial epicondyle hypertrophy, and “having experienced the COVID-19 pandemic” emerged as the only independent predictor. These findings suggest that for developing athletes, the risk of injury may be heightened not only by “excessive throwing” but also by marked fluctuations in throwing activity, such as prolonged periods of limited throwing followed by a rapid increase in throwing volume and load, as seen during the COVID-19 pandemic. Therefore, from a preventive standpoint, carefully managing mechanical load to avoid both overuse and abrupt overload during the growth period may be important.

From a public health perspective, the present findings highlight that structural elbow adaptations in adolescent athletes may be influenced not only by cumulative workload but also by societal or environmental disruptions that alter training patterns at a population level. These findings emphasize the need for individualized and gradual return-to-throwing programs for both pitchers and fielders, especially during adolescence, when the elbow is highly responsive to load fluctuations.

Finally, there are several limitations to this study that need to be addressed. Firstly, the study population was restricted to Japanese high school baseball players. This is a crucial point as the extent of social restrictions and the conditions for resuming sports activities during the COVID-19 pandemic differed significantly across countries. In Japan, relatively strict restrictions on activities within educational institutions, public facilities, and sports organizations were enforced over an extended period. Therefore, caution is warranted when generalizing the findings of this study to other countries. Furthermore, the suppression of baseball activities under such strict restrictions may have influenced bony morphological development during growth, suggesting that regulating pitching frequency could play an important role in shaping elbow joint structure in developing athletes.

Secondly, because this was an observational study, no direct causal relationship could be established between exercise restrictions during the COVID-19 pandemic and structural abnormalities of the elbow joint. Nevertheless, logistic regression analysis was conducted to adjust for potential confounding factors, and the results support the possibility that the COVID-19 pandemic had an independent effect on medial epicondyle hypertrophy. Although causality cannot be definitively established, our supplementary questionnaire lends further support to this interpretation. This study did not follow participants for injury occurrence after entering high school; therefore, no direct association between structural findings and subsequent injury incidence can be determined.

Thirdly, information such as medical history and playing experience was partially based on self-reports, the accuracy of which may be limited. In the future, multicenter collaborative studies that include more detailed tracking of pitching records and activity history will be necessary.

Fourthly, no detailed assessments of physical activity levels during the COVID-19 pandemic were conducted. However, a supplementary questionnaire targeting a subgroup of athletes provided additional insight into changes in practice volume, frequency, and intensity, reinforcing the interpretation that abrupt reloading may have contributed to hypertrophy. Moreover, because the high school baseball team in this study consistently recruits players from junior high school teams of similar competitive levels across Japan each year, we presupposed that, regardless of the presence or absence of the COVID-19 pandemic, there would be no major differences in pre-enrollment practice time or pitching volume between the 2 groups. This suggests that the pandemic probably introduced a substantial disparity in training environments between the groups. Conversely, a notable strength of this study lies in its use of large-scale, directly measured data from 354 high school baseball players situated in their developmental stage. This facilitated a detailed analysis of epidemiological trends in elbow joint imaging findings. The use of a standardized ultrasound evaluation protocol for all participants, combined with assessments conducted by experienced specialists, ensured high consistency and reliability of the data. Our evaluation protocol was conducted by an elbow specialist with 27 years of clinical experience and a physical therapist with over 10 years of experience performing ultrasonographic assessments of baseball players' elbows. This expertise strengthens the validity of the imaging data. Moreover, this study is one of the few investigations in the field of sports orthopedics to elucidate the impact of an unforeseen and sudden societal restriction, such as the COVID-19 pandemic, on musculoskeletal structural adaptations. In addition, exposure to the pandemic was a temporally determined event that occurred independently of individual intention. Although this study utilized a cross-sectional design, the quasi-randomized nature of the exposure provides a unique opportunity to infer a meaningful causal relationship. Therefore, the findings of this study provide valuable insights for reconsidering future injury prevention strategies and pitching load management.

## Conclusion

This study identified a contrasting pattern of structural findings in high school baseball players who experienced the COVID-19 pandemic during their growth period, a pattern characterized by an increased prevalence of medial epicondyle hypertrophy and a decreased prevalence of UCL abnormalities. These findings suggest that abrupt changes in pitching load may be differentially related to bone and ligament structures, providing vital evidence for the necessity of appropriate pitching load management during the growth period.

## Disclaimer:

Funding: No funding was disclosed by the authors.

Conflicts of interest: The authors declare that no financial remuneration related to the subject of this article has been received by any author or any member of their family.
